# Improving Performance of the Human Pupil Orbit Model (HPOM) Estimation Method for Eye-Gaze Tracking

**DOI:** 10.3390/s22239398

**Published:** 2022-12-02

**Authors:** Seungbong Lee, Jaehoon Jeong, Nahyun Kim, Manjae Shin, Sungmin Kim

**Affiliations:** 1 Department of Medical Biotechnology, Dongguk University Biomedi Campus, 32, Dongguk-ro, Ilsan dong-gu, Goyang-si 10326, Republic of Korea; 2 Medical Device Industry Program in Graduate School, Dongguk University, 30, Pildong-ro 1-gill, Jung-gu, Seoul 04620, Republic of Korea

**Keywords:** eye-tracking, eyeball model, model fitting method, eye-gaze tracking, HPOM

## Abstract

Eye-gaze direction-tracking technology is used in fields such as medicine, education, engineering, and gaming. Stability, accuracy, and precision of eye-gaze direction-tracking are demanded with simultaneous upgrades in response speed. In this study, a method is proposed to improve the speed with decreases in the system load and precision in the human pupil orbit model (HPOM) estimation method. The new method was proposed based on the phenomenon that the minor axis of the elliptical-deformed pupil always pointed toward the rotational center presented in various eye-gaze direction detection studies and HPOM estimation methods. Simulation experimental results confirmed that the speed was improved by at least 74 times by consuming less than 7 ms compared to the HPOM estimation. The accuracy of the eye’s ocular rotational center point showed a maximum error of approximately 0.2 pixels on the *x*-axis and approximately 8 pixels on the *y*-axis. The precision of the proposed method was 0.0 pixels when the number of estimation samples (ES) was 7 or less, which showed results consistent with those of the HPOM estimation studies. However, the proposed method was judged to work conservatively against the allowable angle error (AAE), considering that the experiment was conducted under the worst conditions and the cost used to estimate the final model. Therefore, the proposed method could estimate HPOM with high accuracy and precision through AAE adjustment according to system performance and the usage environment.

## 1. Introduction

Eye-gaze direction-tracking technology is a human-computer interaction tool that is used to control machines such as computers. This technology is being studied in fields such as education, engineering, gaming, and medicine [1,2,3,4,5]. Research has been conducted to identify or train reality-based users’ behaviors by applying it to automobiles, wheelchairs, and augmented reality [6,7,8]. However, research on eye-gaze direction-tracking is not limited to recognition and the analysis of physical changes in eye movement. Recently, the scope of eye-gaze direction-tracking studies was expanded to the understanding of human psychology, behavior, emotions, and intentions [8,9]. Thus, eye-gaze direction-tracking studies commonly require the stability, accuracy, and precision of eye-gaze direction detection, as well as fast response speed. If these performance requirements are not satiated, results different from the intended purpose can occur.

The movement of the eyeball is well known as a rotational movement based on the rotational center point. However, studies have shown that the center point of the eye model is inconsistent with the eye’s ocular rotational center point (ORCP) [10,11]. The ORCP is not only useful for calculating the gaze direction angle but also for improving the accuracy of techniques that require spatial analysis, such as the recognition of objects located in the gaze direction and the facial pose. The ORCP should also be considered essential for accurately calculating the eye-gaze direction, considering that the size and rotation radius of the eyeball is different for each person. Based on this, we proposed an estimation method of the human pupil orbit model (HPOM) for eye-gaze tracking. We conducted a study to predict the movement orbit of the pupil as a spherical shape based on various mathematical principles and performed simulation experiments of HPOM estimation. Through this study, we presented a novel model called the HPOM, which enables a simplified eye-gaze direction calculation process in a 3D space using a quadratic equation. Accordingly, the eye-gaze direction calculation could be made using only the pupil center point after estimating the HPOM. As a result of simulation experiments, HPOM has been obtained in real-time by taking an estimation time of less than 1 s with an error of less than 1 pixel. In the HPOM estimation study, the minimum difference of gradient (MDG), an independent variable proposed for real-time HPOM estimation, effectively reduced the load and processing time to suit the purpose. However, it was confirmed that the MDG affected the precision due to the lack of discrimination power [12].

In various studies, the method of detecting the eye-gaze direction is based on the phenomenon that the minor axis of the elliptical-deformed pupil always points toward the rotational center [13,14]. In addition, in studies of detecting the iris region uniformly and the gaze direction detection using artificial intelligence, the minor axis of the pupil, which has been changed to an ellipse, is always directed toward the rotational center [15,16,17]. Moreover, we have demonstrated through simulation experiments that the shape of the pupil appears and rotates in an elliptical shape depending on the eye-gaze direction in an HPOM estimation study [12]. In the simulation experiment, the three-dimensional eye-gaze direction was shown as two angles: the rotation angle of the eye (RAE) and the rotation direction angle of the eye (RDA). In particular, the minor axis of the elliptical-deformed pupil was found to be equal to the RDA. For theoretical verification of this phenomenon, results of simulation by fixing the eye-gaze direction, changing the shooting angle, and measuring have also been reported [18]. In addition, clinical studies on mice and humans have been conducted to confirm the actual measurement error [19,20,21].

For the purpose of resolving the lack of precision caused by the lack of discrimination power of the MDG proposed in the HPOM estimation study and reducing the system load using the fact that the minor axis of the elliptical-deformed pupil is the same as the RDA, this study aimed to improve the speed following decreases in the system load and the precision of the HPOM estimation. Since the method proposed in the HPOM estimation study can be used as a preprocessing process, accuracy and precision errors can affect eye-gaze direction detection [12]. In addition, a small system load and a short time are required as a real-time adaptive gaze direction detection model. Therefore, we propose a novel ORCP estimation method to avoid using the MDG, and the entire process and conditions follow the HPOM estimation study [12]. The proposed method includes the period from after acquiring the pupil shape to before making the final eye-gaze direction detection.

## 2. Materials and Methods

### 2.1. Problems of a Previous Study

We had estimated a pupil’s three-dimensional spatial position by analyzing the projected pupil onto a camera (PPC) observed as an ellipse. In addition, by analyzing a projection relationship between the pupil’s 3D position and the camera, we had obtained HPOM described by the pupil’s rotational radius and the ORCP. Furthermore, we suggested the MDG which is an independent variable to improve system overload, which is expected in the ORCP inference process [12]. The MDG decreased HPOM estimation time by effectively reducing the system load. However, the result showed that the degree of precision was lowered in proportion to the set MDG value. This means that the discrimination power of the MDG is insufficient to ensure consistent positioning of the ORCP. Thus, we propose a novel ORCP estimation method that does not apply an MDG.

### 2.2. Theoretical Background

**Figure 1:**
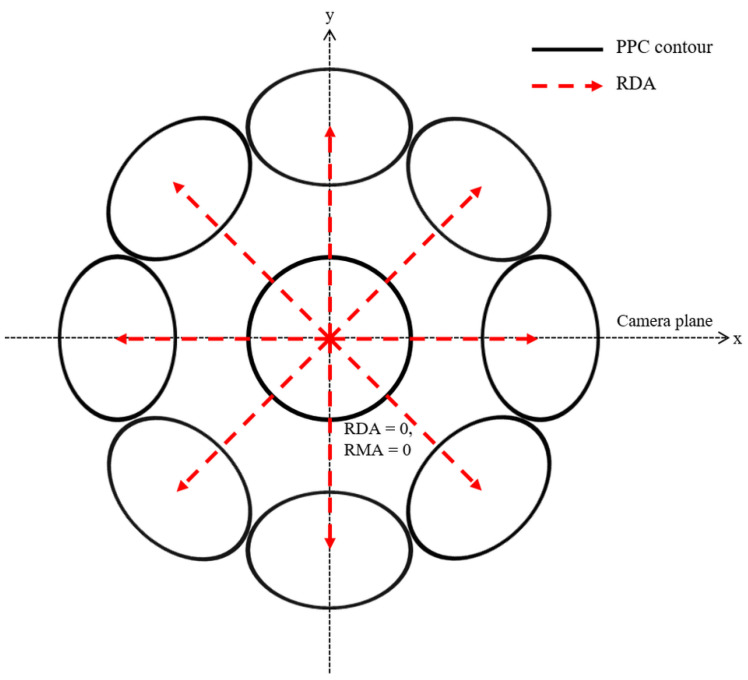
Conceptual diagram of an ideal PPC according to RDA changes.

Figure 1 shows an ideal PPC according to the eye-gaze direction commonly suggested by various studies on eye-gaze tracking, including the HPOM estimation study [12,13,14,15,16,17,18,19,20,21]. The minor axis of the elliptically deformed PPC appears at the same angle as the RDA. This means that the ORCP always exists on the extension line of the PPC minor axis.

**Figure 2:**
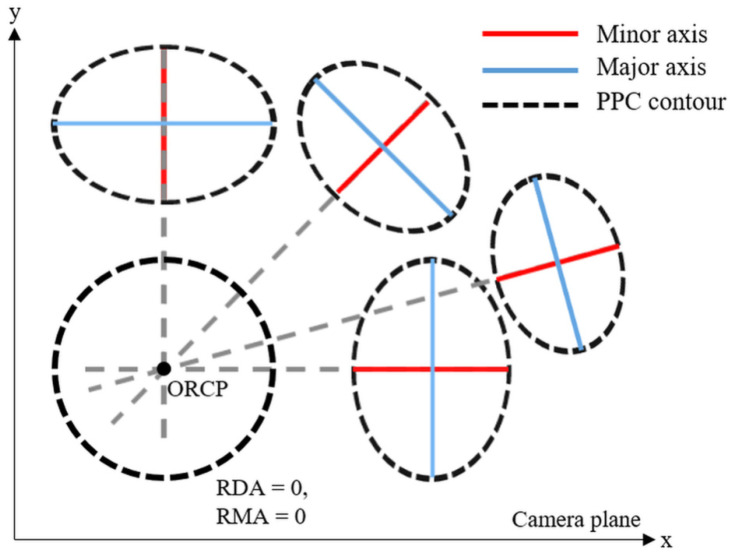
Conceptual diagram of ORCP positioned on the extension line of the minor axis of the PPC.

Figure 2 shows that the ORCP is always located on the extension line of the minor axis of the ideal PPC. In addition, it can be seen that the ORCP is located at the intersection of all extension lines. Based on this phenomenon, the ORCP can be obtained by deriving the equation of a straight line from each PPC.

### 2.3. ORCP Inference

**Figure 3:**
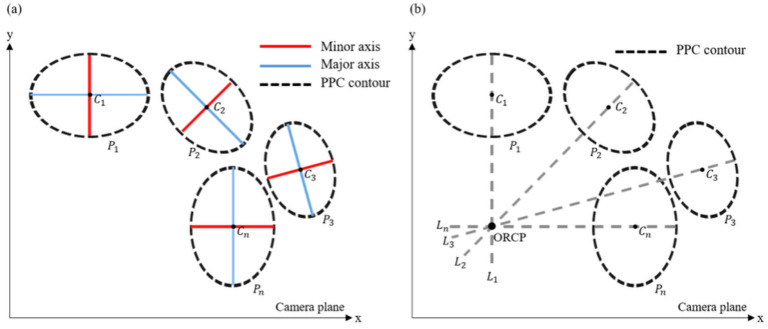
Conceptual diagram of ORCP inference method. (**a**) Obtained PPC data, (**b**) Straight line extracted from PPC.

The PPC contour obtained using the camera is presented in Figure 3a. The center point (Cn) and the direction of minor axis of each pupil (Pn) appears differently depending on the eye-gaze direction. To obtain a straight-line equation from each pupil, the pre-process is required similar to the PPC analysis. A PPC analysis can be performed to obtain the center point, lengths of the major axis and the minor axis, and the rotation angle using the method proposed by Wenchao Cai et al. [22]. In this paper, the rotation angle means the angle forming at the minor axis of a PPC with the *x*-axis. That is, the rotation angle is equal to the RDA. Before transforming the obtained data into the equation of a straight line, the rotation angle must be transformed into a slope. The reason is that equations of straight lines generally express linear change as a ratio. The obtained rotation angle can be calculated as a slope value according to Equation (1). In addition, using coordinates of the center point and the slope value, the equation of a straight line can be obtained as in Equation (2). If Equation (2) is rearranged for an unknown *y*, the equation can be arranged as shown in Equation (3):

(1) Gradient=tan(RDA)

(2) $$\left(y-y_{c}\right)=G\times \left(x-x_{c}\right),\;G=Gradient$$

(3) $$y=Gx-Gx_{c}+y_{c},\;G=Gradient$$

Figure 3b shows a straight line (Ln) obtained from each PPC. One straight-line equation can be obtained from each PPC. Furthermore, since all straight lines intersect in the ORCP, there is always one solution that satisfies the equation of all straight lines. Therefore, the ORCP can be estimated using two samples as minimum limits. However, at least three samples shall be used for accuracy, as an error sample may be included. The position of the ORCP can be obtained through a matrix calculation that consists of straight-line equations. The equation of a straight line can be arranged in a general form, as shown in Equation (4) from Equation (3). If the equation of n straight lines is divided into the coefficient term and unknown term and arranged as a determinant, it can be expressed as Equation (5). The ORCP which expressed as an unknown term can be calculated by finding the inverse matrix of the coefficient term and transforming it into Equation (6). However, since the obtained matrix is not always a square matrix, it is not possible to directly calculate the inverse matrix. Therefore, it is necessary to obtain the inverse matrix using the singular value decomposition method, which can calculate a pseudo-inverse matrix to replace the inverse of a non-square matrix [23].

(4) Ax+By=C

(5) $$\left(\begin{array}{cc} A_{1} & B_{1}\\ \vdots & \vdots \\ A_{n} & B_{n} \end{array}\right)\left(\begin{array}{c} x\\ y \end{array}\right)=\left(\begin{array}{c} C_{1}\\ \vdots \\ C_{n} \end{array}\right)$$

(6) $$\left(\begin{array}{c} x\\ y \end{array}\right)={\left(\begin{array}{cc} A_{1} & B_{1}\\ \vdots & \vdots \\ A_{n} & B_{n} \end{array}\right)}^{-1}\left(\begin{array}{c} C_{1}\\ \vdots \\ C_{n} \end{array}\right)$$

## 3. Experiments

**Figure 4:**
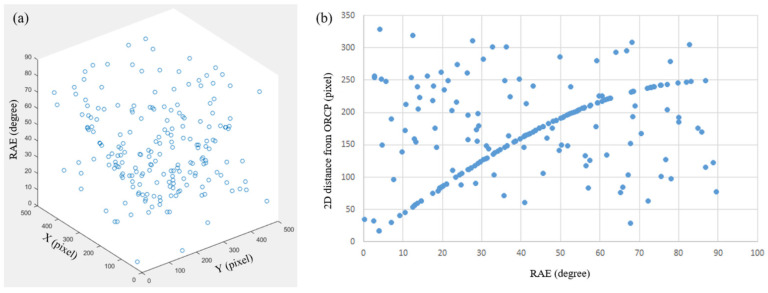
(**a**) Three-dimensional data distribution diagram. (**b**) Data distribution diagram represented by RAE and the distance of the each data point from ORCP on the *xy*-plane.

In this study, a simulation experiment was performed using a total of 200 data points adapted from the HPOM estimation study [12]. The data consisted of 100 ideal PPCs acquired by simulation and 100 randomly configured PPCs as noise. Figure 4a shows the data distribution in the three-dimensional space. The ORCP of the ideal data was set to coincide with the center of the set space. Figure 4b shows the data distribution with the xy-plane distance between the ORCP and each data point and the RAE. The ideal data are observed as a sine wave, as shown in Figure 4b [12]. HPOM estimation is performed using independent variables, model estimation sample (ES), and allowable angle error (AAE), using the random sample consensus method [12,24]. Independent variables ES and AAE refer to the number of samples and tolerances used when estimating HPOM, respectively. The random sample consensus method shows a stable performance in estimating the model indicated by the total data using a small number of ESs. The experiment was divided into two parts to evaluate the effect of each independent variable, AAE and the number of ES, on performance. Each experiment evaluated the performance by comparing the proposed method to the HPOM estimation study [12]. However, the comparison in the second experiment used an MDG of 1 because the discriminatory power decreased as the MDG increased.

In the first experiment, the AAE is fixed to ±5 for comparison with the HPOM estimation performance according to ES. The first experiment was conducted 200 times each, gradually increasing the number of ESs from 2 to 14. Performance comparison and stability evaluation of the newly proposed ORCP estimation method were performed in the second experiment, which was conducted 200 times by fixing the number of ESs to 7 and increasing the AAE by 1 degree from 0 to ±5 degrees.

## 4. Results

The experiment was performed on a computer configured with Intel Core i7 and SAMSUNG 64 GB RAM using the same visual studio 2017 and Open Source Computer Vision Library as the HPOM estimation study [12]. Table 1 shows the results of the first experiment comparing the HPOM estimation study to the proposed method. In the method proposed in the first experiment, the processing time increased proportionally to the number of ESs. When the number of ESs was 4 or less, a processing time of 1 ms or less was required. In addition, the position of the *x*-axis of the ORCP showed an error of less than 1 pixel, while the position of the *y*-axis showed an average error of up to 9 pixels. Moreover, the radius showed an error of less than 1 pixel. When the number of ESs was 7 or less, the standard deviation of the ORCP and radius was constant at 0.

Table 2 shows results of the second experiment comparing the HPOM estimation study to the proposed method. As a result of the second experiment, the proposed method took less than 7 ms of processing time. In addition, as the AAE increased, the error of the ORCP and radius increased. However, the standard deviation of the ORCP was constant at 0.

## 5. Discussion

The HPOM estimation method and the proposed method showed significant differences in the first experiment. With the HPOM estimation method, HPOM could be estimated using at least 4 ESs, whereas the proposed method was able to estimate using at least 2 ESs. In addition, when the number of ES was 4 to 13, the HPOM was estimated approximately 490 to 96,000 times faster, respectively. This means that the system load of the proposed method is significantly less compared to the HPOM estimation method. In addition, the ORCP of the proposed method was found to have a large error compared to the HPOM estimation method. Compared to the HPOM estimation method, the proposed method showed an error of approximately 0.2 pixels on the *x*-axis and approximately 8 pixels on the *y*-axis in the accuracy of the ORCP. The ORCP error is important because it causes eye-gaze direction errors. In addition, it may have a continuous effect on the technology that utilizes the eye-gaze direction detection results. However, this error is thought to be the result of setting a relaxed AAE because the second experiment confirmed that this error was significantly reduced when the AAE was adjusted. On the other hand, the precision of the proposed method was 0.0 pixels when the number of ES was 7 or less, showing a more consistent result compared to the HPOM estimation method. As for the radius, the HPOM estimation study and the proposed method showed an error of less than 1 pixel [12]. It was considered that there was a difference in sensitivity to the AAE applied equally in both experiments. This was because the cost used to estimate the final HPOM was larger than the proposed method compared to the HPOM estimation method. The cost means the number of data when estimating the final model or the number of data forming the model.

As a result of the second experiment, the proposed method compared to the HPOM estimation method, when the MDG was set to 1, was confirmed to be approximately 74 times faster when the AAE was 1 and approximately 78 times faster when the AAE was 5. This proves that the system load of the proposed method is significantly less compared to the HPOM estimation method confirmed in the first experiment. This is because, despite the fact that the purpose of the MDG is to reduce system load, the proposed method showed a markedly faster speed [12]. The accuracy of the ORCP and radius are higher with the HPOM estimation method. On the other hand, the precision of the proposed method was higher than that of the HPOM estimation method. This result can be attributed to the cost. The proposed method generally maintains a high cost compared to the HPOM estimation method. The error ratio of the data should also be considered because using the random sample consensus method explained that if this ratio exceeded 50%, the model could not be secured [24]. Therefore, considering that the experiment was conducted under the worst conditions, the proposed method was judged to work conservatively against the AAE compared to HPOM estimation studies.

Two experiments confirmed that the proposed method could estimate at least 74 times faster than the HPOM estimation method. In addition, the proposed method could obtain a high-precision ORCP and radius. On the other hand, the proposed method shows that the accuracy decreased as the AAE increased because of the conservative tendency compared to the HPOM estimation method [12]. However, when the AAE was 0, the proposed method was ideally able to obtain HPOM. Consequently, stable HPOM estimation is expected to be possible if the AAE is adjusted according to the system performance and environment.

## 6. Conclusions

This study was conducted to improve speed by decreasing system load and the precision of HPOM estimation that is decreased by the MDG. In this study, we proposed a novel ORCP estimation method by reflecting the phenomenon that the minor axis of the elliptical-deformed pupil always pointed toward the rotational center mentioned in various eye-gaze tracking studies. As a result, the proposed method consumed less than 7 ms and reduced the time required by at least 74 times compared to the HPOM estimation study. In addition, compared to the HPOM estimation studies, it showed high precision and low accuracy. However, the proposed method was judged to work conservatively against an AAE, considering that the experiment was conducted under the worst conditions and the cost used to estimate the final model. Therefore, the proposed method could estimate the HPOM with high accuracy and precision by adjusting the AAE according to system performance and the usage environment. In conclusion, the proposed method could constantly estimate the HPOM during a real-time of less than 7 ms. Thus, the purpose of improving speed and precision was achieved. Therefore, the proposed method and the HPOM estimation study can be used as an adaptive model and a preprocessing process for eye-gaze direction detection. In addition, the proposed method is expected to be utilized in various fields not confined to the field of eye-gaze tracking. For example, it is expected to be used for the circular marker-based 3D posed estimation of tools that undergo rotational movements.

## Figures and Tables

**Table 1:** Performance of HPOM estimation according to the number of ES.

	ES	Processing Time	ORCP			Radius	Cost
			X	Y	Distance		
		AVG ^1^ (±SD ^2^)	AVG (±SD)	AVG (±SD)	±SD	AVG (±SD)	AVG
Previous Method	4	82737.40 (4977.1)	250.04 (0.0052)	250.12 (0.0130)	0.01	249.76 (0.0385)	105.58
	5	81188.50 (2952.3)	250.04 (0.0016)	250.12 (0.0049)	0.01	249.76 (0.0223)	105.81
	7	82808.70 (2450.4)	250.04 (0.0008)	250.12 (0.0025)	0.00	249.77 (0.0137)	105.95
	10	85940.50 (2993.2)	250.04 (0.0001)	250.12 (0.0004)	0.00	249.77 (0.0112)	106.01
	14	473,015.30 (15,934.7)	250.04 (0.0004)	250.12 (0.0013)	0.00	249.75 (0.0354)	106.11
Proposed Method	2	0.22 (0.4153)	249.75 (0.0000)	242.05 (0.0000)	0.00	249.87 (0.0000)	109.00
	3	0.42 (0.4940)	249.75 (0.0000)	242.05 (0.0000)	0.00	249.87 (0.0000)	109.00
	4	0.86 (0.3804)	249.75 (0.0000)	242.05 (0.0000)	0.00	249.87 (0.0000)	109.00
	5	1.64 (0.5017)	249.75 (0.0000)	242.05 (0.0000)	0.00	249.87 (0.0000)	109.00
	7	6.54 (0.5921)	249.75 (0.0000)	242.05 (0.0000)	0.00	249.87 (0.0000)	109.00
	10	56.21 (3.8718)	249.76 (0.0141)	242.26 (0.7166)	0.72	249.98 (0.1688)	109.40
	14	960.64 (35.5290)	249.78 (0.0066)	241.50 (0.8077)	0.81	249.85 (0.1529)	110.74
	pcs ^3^	ms ^4^	pixel	pixel	pixel	pixel	pcs

^1^ Average. ^2^ Standard deviation. ^3^ Pieces. ^4^ Millisecond.

**Table 2:** Performance of HPOM estimation according to AAE.

	AAE	Processing Time	ORCP			Radius	Cost
			X	Y	Distance		
		AVG ^1^ (±SD ^2^)	AVG (±SD)	AVG (±SD)	±SD	AVG (±SD)	AVG
Previous Method (MDG 1)	1	435.49 (54.33)	250.04 (0.01)	250.24 (0.60)	0.60	249.76 (0.19)	99.01
	2	427.34 (29.90)	250.03 (0.02)	250.27 (0.31)	0.31	249.76 (0.10)	100.75
	3	427.17 (22.75)	250.03 (0.02)	249.62 (2.26)	2.25	249.67 (0.39)	101.71
	4	465.78 (50.35)	249.83 (0.82)	250.05 (4.10)	4.17	249.81 (0.67)	103.94
	5	530.81 (26.36)	250.09 (0.00)	250.00 (0.78)	0.77	249.74 (0.14)	105.89
Proposed Method	0	4.44 (0.61)	250.00 (0.00)	250.00 (0.00)	0.00	250.00 (0.00)	100.00
	1	5.74 (0.64)	249.97 (0.00)	248.33 (0.00)	0.00	249.92 (0.00)	101.00
	2	5.99 (0.63)	249.97 (0.00)	248.33 (0.00)	0.00	249.92 (0.00)	101.00
	3	6.23 (0.55)	249.97 (0.00)	248.33 (0.00)	0.00	249.92 (0.00)	101.19
	4	6.63 (1.05)	249.86 (0.00)	241.85 (0.00)	0.00	249.89 (0.00)	105.00
	5	6.75 (0.69)	249.75 (0.00)	242.05 (0.00)	0.00	249.87 (0.00)	109.00
	±degree	Ms ^3^	pixel	pixel	pixel	Pixel	Pcs ^4^

^1^ Average. ^2^ Standard deviation. ^3^ Millisecond. ^4^ Pieces.

## Data Availability

Not applicable.
